# A Phase-Gated Protocol for Managing Mid-study Principal Investigator Transitions in Multi-site Investigational Device Exemption Trials

**DOI:** 10.7759/cureus.115098

**Published:** 2026-08-24

**Authors:** Sajan Sanjay Pandya

**Affiliations:** 1 Regulatory Affairs, Encore Medical Inc., Burnsville, USA

**Keywords:** clinical trial governance, investigational device exemption, principal investigator transition, quality by design, regulatory compliance, site management, structural heart devices

## Abstract

Multi-site clinical investigations of high-risk medical devices conducted under the United States Investigational Device Exemption (IDE) framework depend on an unbroken chain of investigator authorization at every active site. When a principal investigator departs mid-study, that chain is tested. A poorly managed transition can interrupt enrollment, leave delegation of authority records out of date, lapse institutional review board (IRB) approvals, and create the documentation gaps that most commonly attract regulatory scrutiny. Despite how routinely these transitions occur in multi-year cardiovascular trials, the literature offers sponsors little practical guidance on how to run one. This technical report describes a phase-gated protocol for managing mid-study principal investigator transitions, developed as the investigator lifecycle component of a broader prospective regulatory governance model for Class III device investigations. The protocol divides each transition into four sequential gates: continuity assessment, successor qualification, authorization transfer, and verification and release. Each gate specifies the documents that must exist, the party responsible for producing them, and the criterion that must be satisfied before the transition may advance. Enrollment at the affected site is governed by an explicit hold-or-continue decision made at the first gate rather than by improvisation. The protocol was applied prospectively during principal investigator transitions in an active, FDA-authorized IDE investigation of a transcatheter structural heart device (ClinicalTrials.gov: NCT05537753), where transitions were completed without enrollment authorization gaps, delegation discontinuity, or IRB approval lapses at the affected sites. As the protocol is constructed entirely from obligations that apply to every IDE sponsor, including 21 CFR Part 812 investigator selection and agreement requirements, financial disclosure under 21 CFR Part 54, and the quality-by-design expectations of ICH E6(R3), it is transferable to any multi-site device investigation. It is offered as a working template that sponsors, particularly those with small regulatory teams, can adopt directly or adapt to local procedures.

## Introduction

Principal investigator turnover is a recurring operational challenge in multi-site clinical investigations. Investigators may retire, relocate, change institutions, or step back from research duties during a study that continues for several years. Under the Investigational Device Exemption (IDE) regulation, the sponsor must select qualified investigators and obtain a signed investigator agreement before permitting them to participate in the investigation [1]. In practical terms, investigator authorization depends on a set of linked regulatory and operational records, including the investigator agreement and qualification documentation, applicable institutional review board (IRB) approval or acknowledgment, financial disclosure documentation, and an effective delegation structure. A principal investigator change does not automatically invalidate every record. Rather, depending on the document type, sponsor procedure, institutional policy, and local IRB requirements, records may require amendment, re-execution, replacement, or reapproval [1,2].

The consequences of an unmanaged transition can extend beyond paperwork. For example, if a principal investigator departs unexpectedly while a site has a subject scheduled for a study procedure or an active follow-up visit, the sponsor must determine promptly which activities may continue, under whose oversight, and whether local IRB action is required. New enrollment may need to pause even while necessary participant follow-up continues under appropriately authorized clinical oversight. If the named investigator, delegation records, and IRB documentation are not aligned with actual site practice, the resulting mismatch may create compliance concerns and weaken the traceability of study conduct [1,3]. Industry surveys have also described substantial regulatory burden and resource constraints within medical-device development, conditions that can make controlled handoffs especially important for smaller sponsors [4,5].

The regulatory environment increasingly emphasizes prospective quality management. ICH E6(R3) asks sponsors to build quality into trial processes and manage risks to participant protection and data reliability proactively [6]. The United States Quality Management System Regulation, effective in February 2026, incorporates ISO 13485:2016 into the device quality-system framework and reinforces the use of controlled, documented processes [7,8].

The protocol described here was developed as the investigator lifecycle component of Integrated Prospective Regulatory Governance (IPRG), a broader operating model for high-risk device investigations [9]. IPRG comprises four components: prospective compliance architecture, cross-functional regulatory integration, investigator lifecycle management, and risk-stratified submission strategy. The present report is intentionally narrower. It focuses only on the investigator lifecycle problem of replacing a serving principal investigator at an active site and describes the operational sequence in sufficient detail to stand independently of the broader model.

Published clinical-trial quality literature provides complementary context for this approach. Quality-by-design work has emphasized identifying critical-to-quality factors before failures occur and embedding controls into trial processes [10]. A structured quality-by-design program has also been shown to be feasible in an academic clinical-research setting [11]. Risk-adapted monitoring studies similarly support focusing oversight on systematic risks rather than relying only on intensive routine checking [12]. Analyses of Good Clinical Practice inspection findings further demonstrate the continuing importance of protocol compliance, documentation, and oversight in regulatory review [13]. The specific operational problem addressed here is narrower than these broader quality-management frameworks: how to convert applicable requirements and sponsor responsibilities into a controlled workflow when a principal investigator changes mid-study.

## Technical report

Design principles and scope

The protocol rests on three design principles. First, a principal investigator transition is treated as a controlled process with defined entry and exit conditions rather than as an informal administrative handoff. Second, each stage has a named owner and a release criterion so that responsibility remains explicit. Third, the enrollment question is addressed at the beginning of the transition rather than after documentation problems emerge.

The four-gate structure was selected because each gate answers a distinct control question: whether study activity can continue during the transition; whether the successor is appropriately qualified and documented; whether authority has been transferred in accordance with applicable sponsor, institutional, and IRB requirements; and whether the transition record is complete enough for the site to return to routine operation. Alternative organizational models could combine or reorder individual tasks. The four-gate sequence, sponsor-defined timing targets, release criteria, and verification checklist therefore represent the author's proposed operational architecture rather than requirements prescribed by the United States Food and Drug Administration (FDA) or the International Council for Harmonisation of Technical Requirements for Pharmaceuticals for Human Use (ICH).

The scope of this report is limited to mid-study replacement of a serving principal investigator at an active site, which is the pathway exercised in live use. The same gate logic may be conceptually adaptable to site activation or closure, but those applications were not evaluated in the reported implementation and are not presented as validated uses of the protocol.

Gate one: Continuity assessment

The trigger is knowledge of an impending or effective principal investigator departure, whether through formal notice from the institution or awareness by the sponsor's clinical team. The sponsor's regulatory lead opens a transition record that becomes the central file for the process. Under the proposed workflow, three questions are addressed within a sponsor-defined target window, typically five business days: the expected departure timeline; the status of enrolled subjects, upcoming study procedures, and follow-up obligations; and whether enrollment or other investigator-dependent activities may continue during the transition.

The hold-or-continue decision is an operational control, not a standalone regulatory requirement. If an appropriately qualified investigator can provide interim oversight and the sponsor, institution, and local IRB requirements permit that arrangement, specified activities may continue under documented coverage. If those conditions are not met or remain unclear, new enrollment is placed on a formal hold, and the decision is documented and communicated. Participant safety follow-up should not be abandoned because enrollment is held; necessary follow-up must continue under appropriately authorized clinical oversight and in accordance with the protocol and local requirements. The purpose of the first gate is to prevent ambiguity about who is responsible for study conduct while the successor pathway is being established [1,6].

Gate two: Successor qualification

This gate assembles the evidence that the incoming investigator is qualified and appropriately engaged before sponsor-defined authority is transferred. Regulatory requirements include sponsor selection of qualified investigators and execution of the investigator agreement under 21 CFR 812.43, together with applicable financial disclosure requirements under 21 CFR Part 54 [1,2]. The proposed operational qualification package also includes current curriculum vitae and licensure information, relevant procedural experience, protocol and device training, and Good Clinical Practice training current under the sponsor's training policy. These items are reviewed against predefined acceptance standards, meaning sponsor-defined criteria used to confirm that required qualification and training records are complete, current, and acceptable before the gate closes.

Qualification activities may proceed in parallel with the continuity assessment when appropriate. Parallel work can shorten the administrative interval, but the workflow does not assume that a successor will be available immediately. If appointment is delayed, the site remains under the activity restrictions established at gate one until compliant coverage or successor authorization is documented.

Gate three: Authorization transfer

Once a qualified successor has been identified, the workflow turns to formal transfer of site authority. The exact sequence must follow the applicable IRB and institutional process. Under the sponsor workflow described here, the IRB submission or amendment naming the successor is initiated early because local IRB action can be a long-lead item. While that action is pending, the delegation of authority record is prepared for the successor, consequential task assignments are reviewed, affected site personnel are retrained where needed, and the sponsor's internal investigator records are updated. The successor's effective authority is not treated as active until applicable IRB approval or acknowledgment and sponsor requirements are satisfied.

A "compliant effective date" in this protocol means an effective date that is not earlier than the required IRB approval or acknowledgment and is consistent with sponsor and institutional requirements. The outgoing investigator's end date is documented, and the transition record is reconciled so that each period of site responsibility can be attributed to a named investigator or documented interim arrangement. This sponsor-defined sequencing should be adapted when local IRB procedures require a different order or documentation set. Separately, 21 CFR 812.150 requires sponsors to maintain FDA reporting obligations, including submission of a current investigator list at six-month intervals [1].

Gate four: Verification and release

The final gate is a deliberate verification of the first three. The sponsor's regulatory lead, or a second reviewer where staffing permits, reviews the transition record against a fixed checklist covering the continuity decision, qualification package, applicable IRB documentation, executed delegation records, investigator-list updates, and written documentation lifting any enrollment hold. The site exits the transition protocol only after the defined checklist is complete.

This verification step is a sponsor-defined operational control. Under 21 CFR 812.150, the sponsor must submit a current list of investigators to FDA at six-month intervals and progress reports at least annually [1]. Recording the principal investigator transition for consideration in the next annual progress report is used in this protocol as a documentation practice to support regulatory traceability; it is not presented as a separate regulatory requirement to narratively report every principal investigator transition in the annual report. "No interval of unattributed responsibility" means that the sponsor's records account for who held documented site responsibility during each relevant period of study conduct.

Implementation experience

The protocol was applied prospectively during three mid-study principal investigator transitions at three affected sites in an FDA-authorized IDE investigation of a transcatheter structural heart device for prevention of recurrent ischemic stroke, registered at ClinicalTrials.gov (NCT05537753) under IDE G220115 [14]. The 2025 IDE annual progress report does not separately enumerate principal investigator transitions or the number of sites affected by such transitions; therefore, transition-specific counts are not reported here.

The overall trial began enrollment on July 12, 2023. At the August 11, 2025 data cutoff, eight sites had been activated, and 13 subjects had been randomized across five investigational sites; these counts remained unchanged at the time of manuscript revision. This trial-level period provides the operational context for the reported experience but should not be interpreted as a measured transition-cycle interval. Transition-specific start and completion dates were not prespecified as evaluation variables.

For this descriptive report, an enrollment-authorization gap was defined as a period in which enrollment proceeded without the sponsor-documented investigator coverage and applicable IRB authorization required by the site workflow; delegation discontinuity was defined as a period in which active study tasks lacked an effective documented delegation structure; and an IRB lapse was defined as a period in which required IRB authorization for the acting principal investigator was not in force. Verification was based on the sponsor transition record and associated regulatory documentation, including IRB records, delegation records, and investigator-list documentation. Transitions managed under the protocol were completed without documented enrollment-authorization gaps, delegation discontinuity, or IRB approval lapses at the affected sites. In each case, the gate one decision was documented within the sponsor-defined target window, and gate four verification was completed before site release.

Across the overall trial, four protocol deviations had been reported through the August 11, 2025 cutoff: two involved out-of-range activated clotting time measurements and two involved timing of follow-up or procedure visits. None concerned principal investigator transition, investigator authorization, delegation continuity, or IRB approval. These trial-wide deviation data provide context but are not a validated performance endpoint for the transition protocol. Transition duration, time to IRB approval, duration or frequency of enrollment holds, transition-specific monitoring findings, audit observations, and corrective and preventive action generation were not prospectively prespecified as performance measures. Accordingly, the implementation experience is presented as evidence of feasibility and operability, not comparative effectiveness. This report presents no patient-level analysis and did not evaluate clinical outcomes attributable to the governance protocol.

Table 1 summarizes the protocol while explicitly separating applicable regulatory or institutional requirements from the sponsor-defined operational controls used to implement them.

**Table 1:** Phase-gate structure of the principal investigator transition protocol. The four-gate sequence, five-business-day target, transition record, acceptance checklist, release criteria, and final verification are proposed operational controls rather than independent regulatory requirements. "Predefined acceptance standards" are sponsor-defined qualification criteria; "compliant effective date" means a date consistent with required IRB, sponsor, and institutional authorization; and "attributable responsibility" means that sponsor records identify the responsible investigator or documented interim coverage for each relevant period. IRB: institutional review board; CV: curriculum vitae; GCP: Good Clinical Practice; IDE: Investigational Device Exemption; FDA: United States Food and Drug Administration.

Gate	Applicable regulatory/institutional requirements	Proposed operational controls	Owner	Release criterion
One: Continuity assessment	Maintain qualified investigator oversight and IRB-compliant site conduct; protect participants and maintain required study records [1,6].	Open transition record; confirm departure timeline; review enrolled-subject status and upcoming procedures; document hold-or-continue decision within sponsor-defined target.	Sponsor regulatory lead, with site coordinator and clinical input	Activity status documented and communicated; interim coverage or enrollment hold clearly established.
Two: Successor qualification	Sponsor selects qualified investigator and obtains required investigator agreement/qualification information; financial disclosure requirements apply [1,2].	Sponsor-defined qualification checklist including CV/licensure, procedural experience, protocol/device training, GCP training, and acceptance review.	Sponsor regulatory lead; incoming investigator	Qualification package complete against sponsor-defined acceptance standards.
Three: Authorization transfer	Applicable IRB/institutional action before successor authority becomes effective; FDA investigator-list reporting obligations continue [1].	Initiate IRB change; prepare revised delegation record; retrain affected personnel; update internal investigator records; document outgoing-investigator end date.	Sponsor regulatory lead; site institution; IRB	Applicable IRB action documented; delegation effective date is compliant; responsibility is attributable for the transition period.
Four: Verification and release	Current investigator list submitted to FDA at six-month intervals and progress reports submitted at least annually under 21 CFR 812.150 [1].	Complete verification checklist; document lift of any enrollment hold; record transition for sponsor reporting/traceability processes.	Sponsor regulatory lead; second reviewer where staffing permits	Every checklist item verified and site formally released from transition workflow.

## Discussion

The contribution of the phase-gated protocol is organizational rather than regulatory. Mandatory elements arise from existing IDE, financial disclosure, IRB, and sponsor reporting obligations, while the four-gate sequence, sponsor-defined timing target, transition record, release criteria, and verification checklist are proposed operational controls. This distinction is important because FDA and ICH do not prescribe this four-gate sequence, and individual sponsors or institutions may use different organizational models.

The rationale for the sequence is to make four failure points visible before a site progresses: uncertainty about whether study activity can continue, incomplete qualification of the successor, premature transfer of authority, and incomplete closeout of the transition record. This is consistent with the quality-by-design principle of identifying critical-to-quality risks prospectively rather than discovering them retrospectively [6,10,11]. Risk-adapted monitoring research similarly shows the value of focusing oversight on systematic trial risks within a broader quality-management approach [12]. In addition, analyses of FDA and European Medicines Agency Good Clinical Practice inspection findings demonstrate that protocol compliance, records, and oversight remain recurring areas of regulatory concern [13]. The present protocol translates those broader quality principles into a narrow operational workflow for principal investigator transitions.

The workflow should not be interpreted as eliminating the practical complexity of a transition. An unexpected departure may leave no overlap period and may require an immediate enrollment hold while the sponsor and institution establish authorized coverage. A delayed successor appointment can prolong that hold. Local IRBs may differ in whether they require prior approval, acknowledgment, specific forms, or institution-specific documentation for a principal investigator change. Sites may also have participants in active follow-up or with scheduled procedures when the transition occurs. In those circumstances, the first priority is to preserve participant protection and required follow-up under appropriately authorized oversight while restricting activities that cannot compliantly proceed. The gate logic is therefore intended to structure decision-making, not to override local requirements.

The protocol may be adaptable beyond the sponsor in which it was developed because the underlying questions of investigator qualification, IRB status, delegation, documentation, and sponsor reporting arise broadly in IDE investigations. Adaptability, however, is a design inference rather than demonstrated generalizability. Successful implementation may depend on staff training, sponsor standard operating procedures, institutional policies, local IRB practices, and available clinical and regulatory resources. Sponsors adopting the framework should therefore map each gate to their own procedures rather than treating the workflow as a universally executable template.

The experience also illustrates the potential value of explicit verification for inspection readiness. A transition can appear operationally complete while one residual record remains inconsistent with the successor's effective authority. Requiring a release checklist creates a deliberate point at which those discrepancies can be found before the site returns to routine operation. This is conceptually aligned with risk-based quality management and inspection-readiness practices, although the present report did not compare this verification step with another method and cannot quantify its incremental effect [10-13].

Several limitations require emphasis. The implementation experience comes from a single sponsor and a single IDE investigation and was managed by the protocol's designer. The exact number of principal investigator transitions and affected sites must be reported from sponsor transition records before final resubmission because those counts are not contained in the annual progress report used for the trial-level context. The reported outcomes are descriptive and have no comparator. Transition cycle time, time to IRB approval, enrollment-hold duration, monitoring findings, audit observations, and corrective and preventive action generation were not prospectively defined as performance measures. The four trial-wide protocol deviations were not transition-related, but they are not a substitute for transition-specific metrics. Site activation and closure were not evaluated under this protocol. Prospective evaluation across independent sponsors, institutions, and IRB environments using predefined process measures would be necessary to assess reproducibility, comparative effectiveness, and generalizability.

## Conclusions

Mid-study principal investigator transitions can be managed using a structured, phase-gated approach that makes continuity, successor qualification, authorization transfer, and final verification explicit. In the reported single-sponsor IDE investigation, the protocol was feasible to operate and transitions were completed without documented enrollment-authorization gaps, delegation discontinuity, or IRB approval lapses at the affected sites. The available evidence does not establish that the protocol is safer or superior to alternative approaches.

The four-gate model should therefore be viewed as a practical operational framework rather than a regulatory mandate or universally validated template. Its use elsewhere may require adaptation to sponsor procedures, staff training, institutional policies, and local IRB requirements. External implementation with prospectively defined measures such as transition duration, IRB turnaround time, enrollment interruption, transition-related deviations, monitoring findings, audit observations, and corrective actions is needed before broader conclusions regarding effectiveness or generalizability can be made.
